# New Applications for Phage Integrases

**DOI:** 10.1016/j.jmb.2014.05.014

**Published:** 2014-07-29

**Authors:** Paul C.M. Fogg, Sean Colloms, Susan Rosser, Marshall Stark, Margaret C.M. Smith

**Affiliations:** 1 Department of Biology, University of York, Wentworth Way, York YO10 5DD, UK; 2 Institute of Molecular Cell and Systems Biology, University of Glasgow, Bower Building, Glasgow G12 8QQ, UK; 3 School of Biological Sciences, University of Edinburgh, King's Building, Edinburgh EH9 3JR, UK

**Keywords:** RDF, recombination directionality factor, IHF, integration host factor, bacteriophages, integrases, genome engineering, integrating vectors, synthetic biology

## Abstract

Within the last 25 years, bacteriophage integrases have rapidly risen to prominence as genetic tools for a wide range of applications from basic cloning to genome engineering. Serine integrases such as that from ϕC31 and its relatives have found an especially wide range of applications within diverse micro-organisms right through to multi-cellular eukaryotes. Here, we review the mechanisms of the two major families of integrases, the tyrosine and serine integrases, and the advantages and disadvantages of each type as they are applied in genome engineering and synthetic biology. In particular, we focus on the new areas of metabolic pathway construction and optimization, biocomputing, heterologous expression and multiplexed assembly techniques. Integrases are versatile and efficient tools that can be used in conjunction with the various extant molecular biology tools to streamline the synthetic biology production line.

## Highlights

• Phage integrases are site-specific recombinases that mediate controlled and precise DNA integration and excision.
• The serine integrases, such as ϕC31 integrase, can be used for efficient recombination in heterologous hosts as they use short recombination substrates, they are directional and they do not require host factors.
• Both serine and tyrosine integrases, such as λ integrase, are versatile tools for DNA cloning and assembly *in vivo* and *in vitro*.
• Controlled expression of orthologous serine integrases and their cognate recombination directionality factors can be used to generate living biocomputers.
• Serine integrases are increasingly being exploited for synthetic biology applications.

## Graphical abstract

**Figure:**
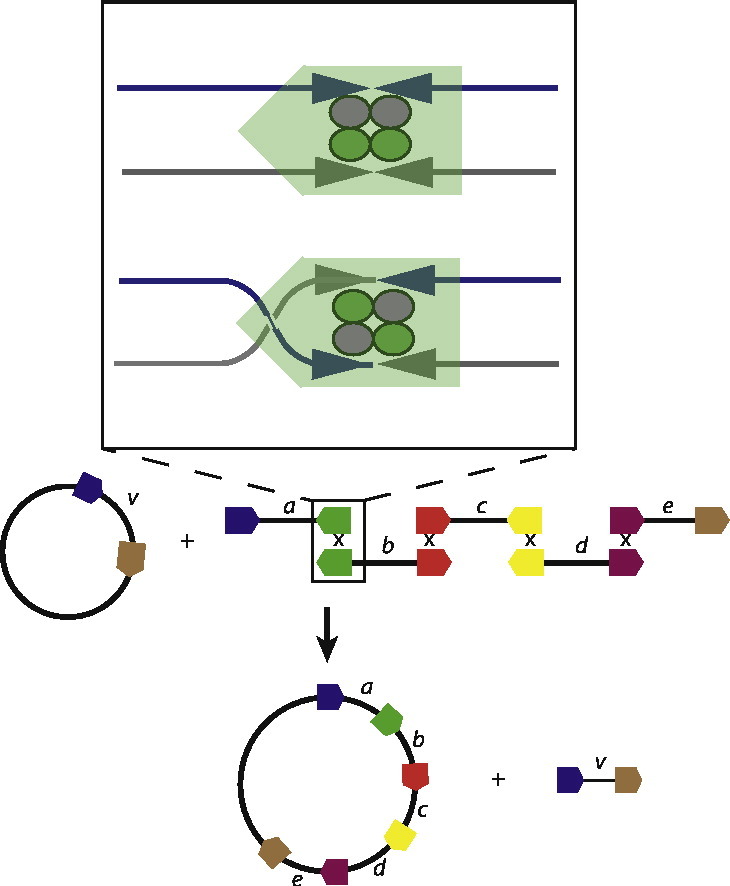

## Introduction

### Bacteriophages

**Fig. 1:**
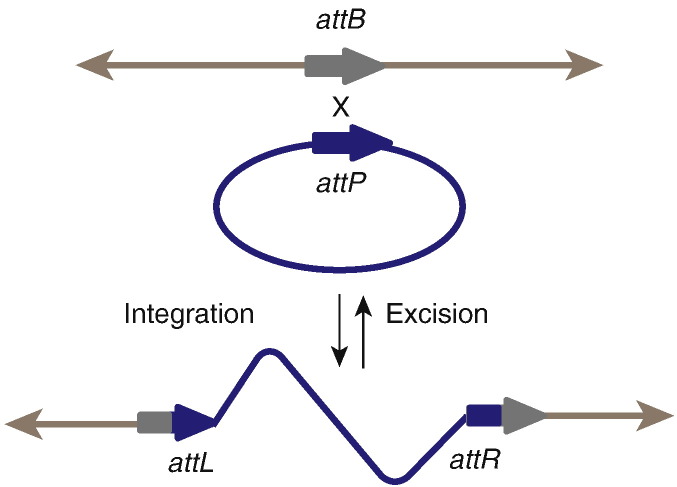
Overview of phage integration and excision. After injection, the phage genome is circularized (blue circle) and can then integrate via the phage attachment site (*attP*; blue arrow) into the bacterial host attachment site (*attB*; grey arrow). The integration reaction produces the prophage flanked by the new attachment sites, *attL* and *attR*, which are hybrids containing half of *attP* and half of *attB*. Excision occurs between *attL* and *attR* to regenerate *attP* on the excised phage genome and *attB* on the host chromosome. Both integration and excision require integrase, the enzyme that mediates the site-specific DNA recombination. Excision also requires a phage-encoded accessory protein, an RDF or a Xis.

Viruses are the most abundant biological entities on this planet, with an estimated 10^30^ present in the oceans alone [1]. The vast majority of viruses are bacteriophages (viruses that infect bacteria) and these outnumber bacteria by about 10 to 1 [2]. Bacteriophage lytic activity significantly affects bacterial mortality and nutrient cycling whereas non-lytic interactions between bacteriophage and host can lead to the distribution of traits beneficial to their hosts, such as antibiotic and phage resistance or increased virulence. Upon infection of a new host, most bacteriophages proceed through the lytic lifecycle, where they subvert the host molecular machinery for the sole purpose of rapid self-replication and dissemination. However, under certain conditions, temperate phages are also able to integrate their own genome into that of the host and remain essentially quiescent for an indeterminate time, until stimulated to return to lytic replication [3]. This process of phage genome integration is mediated by phage-encoded integrases that, in the absence of any other phage-encoded factors, catalyse unidirectional and highly site-specific recombination reactions (Fig. 1). Upon induction back into the lytic lifestyle, phage-encoded accessory proteins known as excisionases (Xis) or recombination directionality factors (RDFs) activate their cognate integrases to mediate the reverse reaction leading to excision of the prophage.

### Conservative site-specific recombination

Site-specific recombination reactions result in the precise integration, excision (or resolution) or inversion of DNA [4]. Despite the different outcomes of recombination, the site-specific recombinases follow a generic pathway. Recombinase binds to two recombination substrates and brings them into close proximity by protein–protein interactions. The substrates are then cleaved and the DNA ends are reorganized in a strand exchange reaction so that rejoining of the DNA backbone gives rise to the recombinant products. An important intermediate during DNA breakage and rejoining is the recombinase covalently linked to the phosphodiester backbone. The formation and removal of the phosphoseryl or phosphotyrosyl bond is energetically neutral, and the reactions do not require an external energy source. This property and the fact that the DNA rearrangements occur without any net loss or gain of DNA are features of conservative site-specific recombination reactions.

There are two evolutionarily distinct families of site-specific recombinase proteins distinguishable by sequence homology and mechanism of action. The families are identified by the eponymous catalytic residue, that is, tyrosine or serine recombinases. Both families of recombinases comprise members that can mediate integration, excision (resolution) or inversion.

### Tyrosine integrases

Integration of the circular phage λ genome into the *Escherichia coli* chromosome by a single crossover was first proposed by Campbell [5]. λ recognizes a specific attachment site in the bacterial chromosome, the *attB* site, and this recombines with a phage attachment site, *attP* (Fig. 1 and Table 1). The result is an integrated prophage flanked by two hybrid sites, *attL* and *attR*, each composed of a half site from *attB* and a half site from *attP*. The integration reaction between *attP* and *attB* is mediated by an integrase, Int, encoded by phage λ. Int, along with Xis, is also required for the reverse reaction in which *attL* and *attR* recombine to regenerate *attB* in the host chromosome and *attP* in a circular phage genome that can re-enter a lytic growth cycle.

**Fig. 2:**
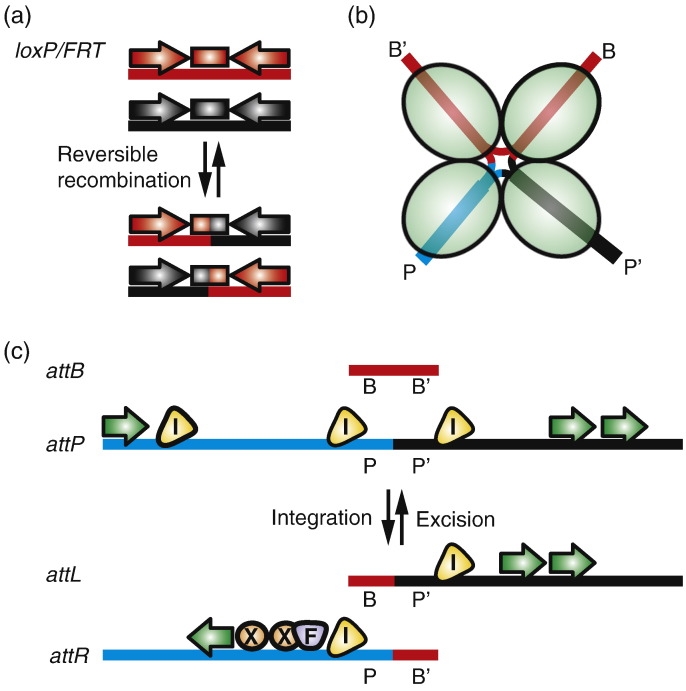
Tyrosine recombinase *att* site structure and the control of integration *versus* excision. (a) Overview of Cre/FLP *att* site requirements. The recombination sites for Cre (*loxP*) and FLP (*FRT*) recombinases are substantially simpler than the λ model. The minimal recombination requirements are two identical 34 bp sites (each substrate here is colour-coded either red or black) composed of 13 bp inverted repeats (arrows) flanking an 8 bp asymmetrical core sequence (boxes) where the crossover occurs [133]. (b) Diagram of the recombination intermediate. During both integration and excision, tyrosine recombinases catalyse pairwise, sequential cleavage and exchange of single DNA strands from the respective *att* sites. This process produces a DNA structure resembling a four-way Holliday junction, which is subsequently resolved to the recombined products. The green ovals represent λ Int subunits that make up the active tetramer. (c) Overview of the important features of the λ Int *attB* and *attP* attachment sites required for effective recombination. Both sites have left and right arms (B- and B′-arms, red lines; P-arm, blue line; P′-arm, black line) separated by a central identical sequence of 15 bp, which contains the site of recombination (data not shown). Here, the *attL*/*R* sites are annotated using the λ convention (*attL* = *BP*′ and *attR* = *PB*′). The *attP* site is approximately 240 bp in length and, in the integration reaction, λ Int binds to the arm binding sites (green arrows), and IHF binds to its cognate sites (yellow). In excision, *attL* has binding sites for λ Int and IHF and *attR* has a binding site for λ Int on the P′-arm, which is in a different position and inverted compared to the site used for Int binding in integration. *attR* also has binding sites for Fis (purple) and Xis (orange).

λ Int is the founding member of the tyrosine recombinase family and has been the subject of intense study [3,6,7]. The recombination mechanisms used by λ Int and a related recombinase, Cre from phage P1, are representative of almost all tyrosine integrases [7]. The recombination substrates for the tyrosine recombinases generally are composed of inverted repeats flanking a short 6 to 8 bp non-palindromic sequence or core sequence that is identical between recombining partners (Fig. 2) [8,9]. Tyrosine recombinases bind to the inverted repeat sequences and bring the two sites together in a tetrameric complex or synapse. The formation of the correct synaptic complex is necessary for activation of the recombinases. The catalytic tyrosine residues in two of the recombinase monomers within the tetramer attack the DNA backbone to produce a covalent 3′ phosphotyrosine enzyme intermediate and a free 5′OH terminated DNA strand. The free OH groups from opposing DNA substrates then displace the covalently bound integrases to create a recombinant joint resembling a Holliday junction (Fig. 2b) [10]. The steps are repeated for the remaining two recombinase monomers and DNA strands, in order to resolve the structure and complete DNA exchange.

The distinguishing feature of the phage integrases is that they are strictly controlled with respect to integration *versus* excision. Unlike Cre and its recombination site, *loxP*, λ Int has four different recombination substrates: *attP*, *attB*, *attL* and *attR* (Fig. 2a and c). Directionality of λ Int recombination is determined by accessory proteins and their binding sites in the substrate DNA, in particular, in *attP* (Fig. 2c). The *attB* site for λ Int resembles the simple recombination substrate described above, that is, an inverted repeat sequence flanking the core. The *attP* site is considerably larger (~240 bp for λ) and more complex; in addition to the core complementary sequence, *attP* contains a series of overlapping regulatory binding sites for integrase and accessory proteins (Fig. 2c and Table 1) [9,11]. The only phage-encoded protein required for an integration reaction is the integrase. However, the host-encoded protein integration host factor (IHF) binds to the *attP* arms and introduces sharp bends that are required for the formation of an integration-competent synaptic complex. Within this complex is a tetramer of integrase bound to the core DNA sequences, derived from *attB* and *attP*. Two sequential DNA cleavage and strand exchange steps occur to generate *attL* and *attR*. Crucially for the maintenance of a stable prophage state, the *attL* and *attR* sites are not substrates for the integrase and IHF alone. In order for the prophage to be excised, integrase, IHF and additional accessory proteins are required, a phage-encoded excisionase (Xis) and host-encoded Fis [12,13]. Together, IHF, Xis, Fis and integrase bind to the P-arm in *attR* inducing a compact loop structure that reconstitutes a tetrameric integrase complex bound to the core sequences that resembles the complex present during integration, but with a modified configuration of the core DNA sequences (Table 1) [10,14]. Sequential DNA cleavage and strand exchange reactions within this excision-competent complex yields *attP* and *attB* once more.

## Applications of Tyrosine Integrases

### Chromosome integration and cloning tools

Integrase-mediated chromosomal integration systems for transgenes were first described in the early 1990s for *E. coli* [15], *Staphylococcus aureus* [16] and Actinobacteria [17–19]. These techniques relied on the use of either a single suicide plasmid encoding a selectable marker gene, *int* and *attP* or a two-plasmid system in which an *attP* is carried on a suicide plasmid and is introduced into target cells that already contain a separate integrase expression plasmid. Site-specific integration occurs via the endogenous *attB* site and can be easily selected for after conjugation or transformation. The integrated plasmids are very stable so long as the *xis* gene, often located adjacent to the phage *int* gene, is not included in the integrating vector [17]. This approach was soon proposed for the production of recombinant vaccines using the *Bacillus* Calmette–Guérin strain widely applied as a live attenuated vaccine against *Mycobacterium tuberculosis* [20]. An integrating plasmid based on mycobacteriophage L5 integrase was constructed with an expression cassette consisting of the promoter region and the first six codons of the major heat shock protein Hsp60 followed by a multiple cloning site. Genes encoding antigens of interest could then be introduced as fusion proteins to be expressed *in vivo* post-vaccination.

These methods were fine-tuned in *Pseudomonas* where integrating vectors that exploited the ΦCTX recombinase were created. In addition to integrase, the plasmids contained the ΦCTX *attP* site, a tetracycline resistance gene and a multiple cloning site [21,22]. Conjugation into *Pseudomonas aeruginosa* produced efficient integration into the native chromosomal *attB* site (frequencies of 10^− 8^ to 10^− 7^) [21]. FRT sites flank the multiple cloning site and facilitate the removal of the entire plasmid backbone upon expression of Flp recombinase, rendering the final recombinant strain markerless an essential feature for any environmental or medicinal applications. Similar single-copy integration vectors have since been constructed for use in other organisms, such as *Francisella tularensis* [23]. An interesting adjunct to this is the recent description of a so-called “Clonetegration” technique, which combines cloning and site-specific integration into a single step to accurately deliver DNA of interest into a native *attB* site [24]. The requisite pOSIP plasmid contains two multiple cloning sites that flank a pUC origin of replication for plasmid propagation and the counterselection marker *ccdB*, both of which are replaced during the initial cloning step by the desired insert using standard techniques, for example, ligation or Gibson assembly. The reaction mix is then directly transformed into the target cells. As pOSIP also contains an antibiotic resistance gene, an *attP* site and a cognate temperature-inducible integrase gene (induced immediately after the DNA is introduced by transformation), integration into a compatible chromosomal *attB* site occurs. The integrase and antibiotic resistance genes are flanked by FRT sites and thus can be removed by expression of Flp recombinase if required. The efficiency of this procedure was tested for five tyrosine integrases endogenous to *E. coli* (ϕ80, λ, HK022, P21 and 186) plus one heterologous serine integrase (ϕC31). Multiple orthologous integrases can also be used to mediate simultaneous or sequential integration of different plasmids into their respective *attB* sites. It is important to note that most of these integration vectors rely on the presence of endogenous *attB* sites and host-encoded factors; therefore, it is a common practice to source the integrase from a phage infecting the bacterial strain intended for genetic manipulation. Whilst convenient, the disadvantage of relying on the endogenous *attB* sites is the lack of control on where the *attB* site is located. One of the recombination systems developed in Clonetegration is the heterologous ϕC31 phage integration system, a serine integrase described below. The ϕC31 *attB* site was inserted at a pre-defined site in the chromosome, thus expanding the integration strategies available to the researcher [24,25].

A key goal for synthetic biology is to be able to mobilise large (megabase pairs down to 100 s of kilobase pairs) fragments of DNA for use in diverse target organisms, many of which may not be amenable to standard molecular genetic techniques. Integrases can provide a means of efficient integration of large DNA fragments into specific genomic locations. For example, the *intB13* λ-like integrase of an integrating conjugative element (ICE*clc*) found in *Pseudomonas knackmussii* has recently been shown to catalyse the targeted integration of large cosmid and BAC substrates (up to 75 kb) into the genome of the related *Pseudomonas putida* species [26,27]. Expression of the integrase in *E. coli* resulted in severe growth inhibition; therefore, integration vectors were constructed with a hybrid promoter containing *lacO* sites to tightly repress integrase expression in *lacI*+ hosts [24,25]. After conjugation into the target *Pseudomonas* species, which does not possess *lacI*, repression is alleviated and efficient integration can occur [26]. In addition, the integration cassette was also incorporated into a *tnpA* mini-transposon that can be mobilised into pre-existing vectors to rapidly produce integrating plasmids without the need for *de novo* construction.

## Gateway™

The most widely used tyrosine integrase recombination system is the Gateway™ cloning method (Life Technologies Ltd, California). Gateway™ is based on an *in vitro* application of λ integrase to facilitate rapid cloning of linear DNA into an “entry vector” and subsequent exchange into specialised plasmids for downstream use [28–30]. Essentially, the system exploits the ability of the integrase to accurately and efficiently rearrange DNA that is flanked by compatible *att* sites; the identity of these *att* sites (*attP*/*B*/*L*/*R*) relative to the insert allows the transfer of target DNA to be precisely manipulated. For example, an insert flanked by two *attB* sites will be swapped with another insert flanked by two *attP* sites when incubated with purified integrase and IHF. Supplementation with excisionase protein will catalyse the reverse reaction to restore the original substrates. Use of a recombinase in this manner removes the problems associated with traditional cloning, for example, reliance on the presence and compatibility of restriction sites, time-consuming reactions and multiple purification steps. This simple application has appeared in hundreds of publications, demonstrating the huge potential for optimization and commercialisation of recombinase technology.

Although useful for relatively straightforward cloning or DNA mobilization applications, there are several major drawbacks of phage-encoded tyrosine integrases. First is a requirement for host-encoded accessory proteins to bend the *attP* substrate to favour efficient integration or excision reactions. Consequently, the *attP* site is long and complex to accommodate multiple integrase and accessory protein binding sites (Table 1). There are rare instances where host accessory proteins are non-essential for tyrosine integrase activity but merely act as subtle enhancers; however, the minimal *attP* sites for these enzymes are still comparable in size to other family members [31,32]. Certain mutant integrases that overcome some, but not all, of these limitations have been developed, but these suffer from severely attenuated efficiency (for review, see Groth and Calos [33]). Furthermore, tyrosine integrases have a strong preference for a supercoiled *attP* site for integration and *attL* and *attR* for excision. The major drawbacks of the tyrosine integrases for complex DNA assembly are therefore the large scar sequences (*attP*, *attL* and *attR*) and the need for host proteins that limit the potential for cross-species or cross-genera use.

### Serine integrases

**Fig. 3:**
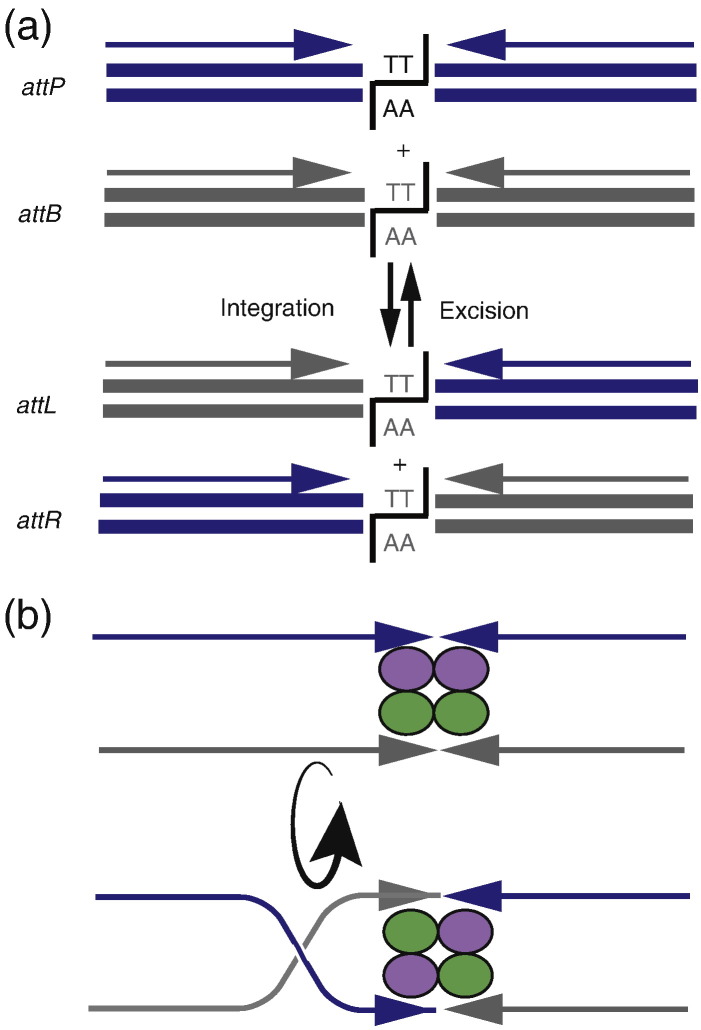
Breakage and rejoining of the attachment sites by serine integrases. (a) Organization of the attachment sites used by the serine integrases. The attachment sites are less than 50 bp in length and *attP* (blue) and *attB* (grey) are inverted repeats. The thick blue and grey lines represent the two strands of DNA with the central dinucleotides shown, in this case, two T:A base pairs. The black lines represent the position of the staggered breaks made when integrase cuts all four strands of the two substrates concertedly. Exchange of the half sites represents the integration reaction (down-pointing arrow) and the excision reaction (up-pointing arrow). The bases in the staggered ends base pair in the recombinant arrangement and integrase can then rejoin the phosphodiester backbone to generate the products. (b) The DNA half sites are exchanged by subunit rotation. Within the active tetramer of integrase subunits, one pair of subunits, still bound to the half sites from opposing substrates, rotate 180° compared to the other pair of subunits. This action brings together the DNA ends originating from different substrates, which can then be joined to form products.

**Fig. 4:**
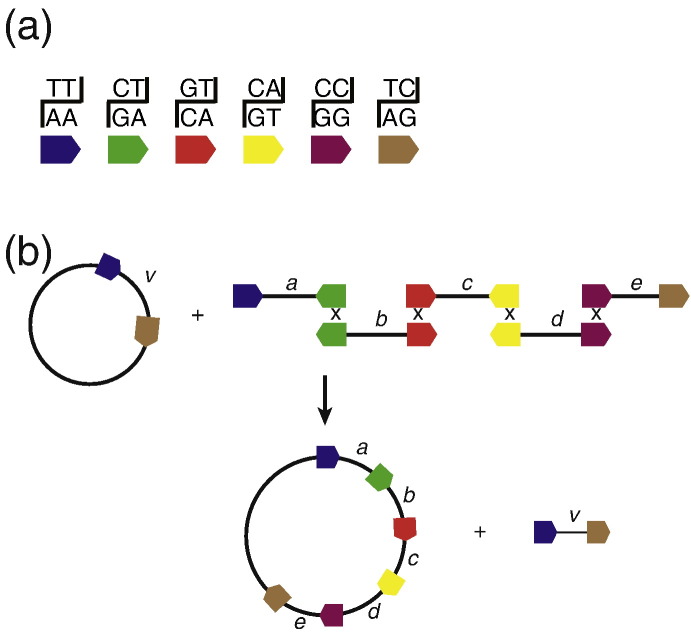
Use of serine integrases for DNA assembly. Attachment sites will only recombine if their dinucleotides involved in the staggered break can base pair in the recombinants (Fig. 3). It is therefore possible to match *attP* and *attB* sites together with identity of the two nucleotide bases at the crossover site. (a) Six possible dinucleotides at the centre of the attachment sites for use with ϕC31 integrase have been colour-coded. Thus, only *attP* and *attB* with the code navy blue will recombine, only those with the code green will recombine and so on (note that *attP* or *attB* sites do not recombine with themselves). (b) Use of the dinucleotide specificity to assembly DNA in a predictable and ordered way. The five DNA fragments shown here encoding five different fragments encoding either single or multiple genes from a metabolic pathway can be recombined together using a single integrase in an *in vitro* recombination reaction. Addition of the sixth fragment containing the vector allows the assembly to be amplified in *E. coli*. Adapted from Colloms *et al.*[101] with permission.

Serine integrases possess a number of properties that overcome some key impediments to synthetic biology offered by their tyrosine integrase counterparts. Perhaps most important is the ability to catalyse recombination between relatively simple attachment sites with no impairment of directionality control [34,35]. Both the *attB* and *attP* sites are typically ≤ 50 bp with different inverted repeat sequences flanking 2 to 12 bp of complementary sequence, which includes the recombination crossover point (Table 2 and Fig. 3a) [36,37]. Serine integrases bind to their target attachment sites as dimers. An exciting recent development is a structural model of a serine integrase bound to its cognate *attP* half site revealing for the first time how integrase recognizes its substrates [38,39]. Interactions between the integrase dimers bound to *attP* and *attB* or *attL* and *attR* form tetramers that bring the recombination crossover sites into close proximity to form the synaptic complex (Fig. 3b) [35,40–42]. Within this complex, integrase is activated and the catalytic serine residues break all four DNA strands simultaneously [35,41,42]. These double-strand breaks are staggered with 2-nucleotide 3′ overhangs and the concomitant formation of a phosphoseryl covalent bond between each integrase monomer and the four recessed 5′ ends [7,41,42]. Each cleaved end remains covalently bound to an integrase monomer as two integrase subunits rotate 180° relative to the other two subunits in a process known as subunit rotation [43–46]. This process effectively swaps two half sites such that ligation of the cleaved ends yields recombinant products (Fig. 3b) [40,46–48]. The complementarity of these core bases in compatible *att* sites is essential for recombination to proceed to completion [40,46,47]. Only when the core bases are the same in the two parent attachment sites can the DNA backbone of the recombinants be joined and this feature is useful for dictating which ends can join to which in a DNA assembly strategy (see below and Fig. 4). It should also be noted that there is no topological restraint on the position of the attachment sites; although *attB* and *attP* are normally located on different DNA molecules in which the outcome is integration, if they are positioned on the same molecule either in the head-to-head orientation or in the head-to-tail orientation, integrase will mediate inversion and deletion respectively.

In phage integration reactions that depend on serine integrases, the only protein required to mediate *attP* × *attB* recombination is the integrase itself, unlike tyrosine integrases that require an accessory host protein (IHF) for integration. Most serine integrases are inert on *attL* and *attR*, although traces of the excision reaction have been observed in some systems [49,50]. This controlled directionality allows integrating plasmids, such as those described above based on the tyrosine integrases, to be extremely stable in the chromosome. For efficient excision to occur, the serine integrases require a phage-encoded RDF. RDFs that work in conjunction with serine integrases have been discovered in several phage recombination systems (Table 1), although only two have been described in any detail biochemically: Bxb1 gp47 and ϕC31 gp3 [51,52]. The RDFs control the directionality of recombination in two ways; first, by enabling synapsis between *attL* and *attR* sites and thus activating excision, and second, by inhibiting synapsis between *attB* and *attP* sites, reducing integration [52]. gp3 and gp47 act by protein–protein interactions with their cognate Int [51,52]. ϕC31 gp3 is able to bind directly to Int both in solution and when Int is complexed with DNA and seems to impart a conformational change in integrase that favours excision [52]. It is worth noting that despite the lack of characterized RDFs, ϕC31 gp3 is closely related to those encoded by the *Streptomyces* phages ϕBT1 and TG1, and there are numerous mycobacteriophages with RDFs related to Bxb1 gp47 [52,53]. The RDFs related to gp3 and those related to gp47 appear to have evolved independently to act as RDFs using similar mechanisms, presumably through convergent evolution [52].

In practice, the minimalist requirements of serine integrases for *att* sites and accessory proteins mean that *in vitro* and *in vivo* strategies for DNA assembly and other applications are far easier to design and optimize. This point is particularly salient when the system is applied to heterologous organisms that may share very little in common with the native host. Furthermore, serine integrases do not require any of the substrates to be supercoiled so that linear fragments can be recombined efficiently *in vitro* in both integration and excision reactions.

## Applications of Serine Integrases in DNA Integration and Genome Engineering

The original application of ϕC31 integrase was in the development of integration vectors for use in *Streptomyces*, which is the natural host for ϕC31 and therefore contains an innate *attB* site for integration [55,56]. These efficient and stable integrating plasmids rapidly became the vectors of choice for introducing DNA into the streptomycetes and related bacteria and can be used for precise ablation of target sequences [57] or even for the transfer of whole antibiotic biosynthesis clusters into heterologous hosts (for reviews, see Refs. [58] and [59]). Serine-integrase-based integration vectors, like those based on the tyrosine integrases, can also be multiplexed within a single organism without any interference between them [60].

### Heterologous hosts

The use of serine integrases in heterologous hosts took hold very quickly after their biochemical properties were first described [61]. The idea of importing site-specific recombination systems into higher eukaryotes to create rational, precise genome rearrangements was first developed with the phage P1 “Cre/*loxP*” and the yeast “Flp/*FRT*” systems, both of which use tyrosine recombinases [62,63]. Both Cre/*loxP* and Flp/*FRT* regenerate the original substrates after recombination. Consequently, both systems are efficient at creating deletion mutants by excising DNA located between directly repeated recombination sites but their reversibility is problematic for stable DNA integration. This problem can be mitigated by recombination site mutations in either of the inverted repeats or the central spacer region (Fig. 2a) that facilitate “Recombinase Mediated Cassette Exchange”, or RMCE (for review, see Turan *et al.* [64]). For example, in the Cre/*loxP* system, even single nucleotide changes within the *loxP* spacer region can lead to incompatibility with the WT (*w*ild-*t*ype) site whilst retaining compatibility with an identical mutant site [65,66]. By flanking target and donor DNA with heterologous *loxP* sites (e.g., *loxP*_mut_-target-*loxP* and *loxP*_mut_-donor-*loxP*), cassette exchange is promoted and unwanted deletion events precluded [66]. The reaction is not obligately unidirectional but can be forced close to completion by a molar excess of the donor [64]. Meanwhile, sites containing certain mutations in either of the left or right inverted repeat sequences are functional alone but double mutants are inactive [67]. These properties have been used independently or in conjunction with the spacer mutants to bias recombination towards integration [66]. Briefly, if the left inverted repeat is mutated in one *loxP* site and the right is mutated on its partner site, recombination will produce a WT site and an inactive double mutant, thus inhibiting further recombination.

The major advantage of the serine integrases is that they can catalyse highly efficient irreversible recombination using simple *att* sites and, for this reason, can also be used for applications that require efficient DNA integration in addition to cassette exchange. Simple modifications have been used to increase serine integrase activity in heterologous systems, for example, the introduction of a nuclear localization signal [68], codon optimization [69], fusion of the integrase ORF (*o*pen *r*eading *f*rame) to an endogenous 3′ untranslated region to favour *in vivo* expression in zebrafish [70] and addition of a 3′ polyadenylate cap to increase mRNA stability in a number of insect model organisms [71–73]. A concept that has rapidly taken root is that ϕC31 integrase can be an important tool for gene therapy, specifically the introduction of corrected genes into a mammalian chromosome through integration into endogenous sites that resemble attachment sites, termed pseudo-*attP* sites [74]. Integration into pseudo-*attP* sites has been used in several medicinally relevant studies, for example, production of therapeutic levels of human factors VIII and IX in mice [75,76], gene therapy of skin disorders by functional complementation of human progenitor cells or primary keratinocytes [77,78], creation of transgenic cattle that express milk laced with the human β-defensin-3 antimicrobial peptide [79] and expression of transcription factors to generate induced pluripotent stem cells from mouse embryonic fibroblasts and human amniotic cells [80]. Mapping of 196 pseudo-*attP* integrations in three human cells lines detected 101 pseudo-*attP* sites that could be used by ϕC31 integrase [81]. Despite these observations, there are problems associated with reliance on pseudo-*attP* sites for site-specific integrations. First, integrations into the pseudo-*attP* sites are often non-reciprocal; that is, small deletions were a frequent occurrence in the flanking DNA [81,82], although small deletions can also occur in mammalian cell lines even when integrases are provided with two cognate attachment sites [83]. Second, it is debatable whether integrase genuinely recognizes the pseudo-*attP* sites or whether these events are simply “ectopic” or random integrations. Either way, the frequency of use of pseudo-*attP* sites is very low as integrase can be expressed constitutively in the presence of one of its attachment sites over many cell duplications without any observable gene rearrangements occurring at the cognate attachment site [82,84,85].

In order to make use of the high efficiency of site-specific recombinases, a docking or landing site containing an attachment site is normally introduced into the chromosome of a cell line or animal that will recombine with the partner attachment site in the presence of integrase. The docking site/landing pad is inserted either randomly into the target genome using established methods of transgenesis or by homologous recombination, as demonstrated in mouse embryonic stem cells or in the chicken DT40 cell line [74,83,84,86]. Transgenes can then be integrated efficiently into the docking site via site-specific recombination. Besides increased efficiency of recombination, there is also the additional advantage that a specific docking site can be identified that supports reproducibly high levels of gene expression and a low likelihood of gene silencing. For example, in mouse embryonic stem cells, a reliable position for the integration of transgenes is the ROSA26 locus [87,88]. A docking site for ϕC31 integrase was introduced into the ROSA26 locus in a mouse line to create an *attP* knock-in. Pronuclear injection of plasmid DNA into zygotes for this mouse line yielded 40% of pups with the desired DNA located in the docking site [86]. This is a highly efficient and reproducible way of generating transgenic mice and the line is marketed under the trade name TARGATT™ (Applied StemCell, Inc).

Docking sites have also been successfully introduced into insects, including *Drosophila* [89] and *Anopheles* [90] species. The use of docking sites in *Drosophila* has had a substantial enabling effect on genetic research in this model organism as the introduction of different alleles at a single position permits greater consistency between constructs and largely avoids transgene position effects [91]. Significantly, the list of organisms in which ϕC31 integrase has been used for genome engineering continues to grow. In addition to the uses in mammalian cell lines and insects detailed above, serine integrases are now being applied in zebrafish [70,86,92] and silkworm embryos [73,93] and, for marker deletion, in a variety of plant species including *Arabidopsis*, barley and wheat [94–96].

Use of multiple site-specific recombinases has become possible since the advent of ϕC31 integrase-mediated genome engineering. An iterative assembly method has been developed in mammalian cells whereby successive rounds of plasmid integration using Cre recombinase are followed by removal of the selection marker and *loxP* site by a serine integrase from either ϕC31 or ϕBT1 [84,85]. As illustration, this iterative technique was used to produce a 400 kb transgene array in a human artificial mini-chromosome in the Chinese hamster cell line [85]. There is no theoretical limit to the number of rounds that could be carried out and entire genomes could be systematically constructed by this method. Monetti *et al*. use a combination of recombinases to increase the range of genome engineering approaches in mice [97]. Their technology is to replace a critical part of a gene with a ϕC31 *attP* docking site creating both a null mutant and the opportunity to reinsert different alleles. The technique uses Flp/FRT to remove unwanted vector and marker sequences and leaves the researcher the option of using the Cre/*loxP* system elsewhere in the genome. This approach has been adopted by the North American Conditional Mouse Mutagenesis Project†, and to date, they have created null alleles in more than 600 genes.

## Use of Serine Integrases in Synthetic Biology: Metabolic Pathway Assembly

Microbes have long been used to manufacture useful transgenic compounds, a successful example of which is artificial human insulin production by *E. coli* [98]. However, the traditional technology of plasmid expression vectors created using restriction enzymes or homologous recombination is only suitable for relatively simple products requiring a couple of genes for synthesis. More complex applications require more sophisticated methods for the construction, mobilisation and optimization of large multi-component synthetic operons [99]. Site-specific recombinases are capable of filling this niche.

The integrase isolated from the *Streptomyces* phage ϕBT1 has been used to assemble *in vitro* DNA fragments encoding the genes required for large and complex secondary metabolic pathways. The use of the integrase-mediated DNA assembly modularizes the pathway such that parts can be easily changed without having to reclone the entire pathway from the beginning [50,100]. The system exploits the requirement for a complementary central dinucleotide pairing between *attB* and *attP* sites in order for recombination to occur, therefore allowing six orthologous target sites. Using this system an ~ 62 kb epothilone synthesis cluster was constructed, including the large *epoD* gene (~ 22 kb), in two construction steps. At least seven fragments could be stitched together in a single *in vitro* reaction.

Independently, using a similar strategy to that of Zhang *et al.* but based on ϕC31 integrase (Fig. 4) [100], directional assembly of up to five components flanked by orthogonal asymmetric *attB*/*attP* sites into a plasmid vector was achieved at high efficiencies [101]. The efficiency and fidelity of even the five-gene assembly was easily sufficient to make downstream applications practical; 18% of > 10^5^ colonies/reaction contained the desired construct. In the recombined product, each component is abutted by *attL* sites, which, when provided with RDF, can recombine with *attR* sites to integrate up to a further five modules or simply to provide flexible removal and replacement opportunities. The utility of this system was demonstrated by the successful integration of an operational, artificial carotenoid biosynthesis operon in *E. coli*. Various enhancements could then be tested by varying gene order, RBS optimization and supplementary gene provisions. Clearly, these new tools could have a major impact on industrial production of biological products, for example, modification of amenable hosts with synthetic operons for conversion of biomass to biofuel or production of novel antibiotics.

## Memory and Counting Devices

**Fig. 5:**
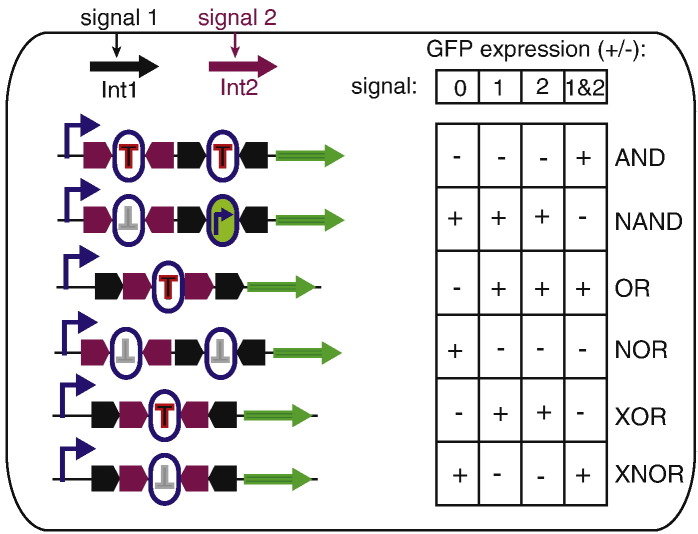
Using integrases to construct Boolean logic gates in *E. coli*. Extracellular signals [1,2] activate the synthesis of two different integrases (Int1 and Int2) that have different cognate and non-cross-reacting recombination sites (shown as either pink or black block arrows). If the recombination sites are oriented head to head, inversion occurs (AND, NAND, NOR, XOR and XNOR gates), but if they are head to tail, deletion occurs (OR gate). Between the recombination sites is either a unidirectional transcription terminator (red T in oval when active; grey inverted T when inactive) or a promoter (black arrow inside the green oval). The green arrow represents a reporter gene such as EGFP. Adapted from Bonnet *et al.*[106] with permission.

Another intriguing application for serine recombinases is to form an integral component of a binary biological processor. The use of recombinases for the creation of biological computers that count and record stimuli has been described using invertases (Hin or FimB) or non-directional tyrosine recombinases (Cre and Flp) [102–104]. The ability to control the direction of serine integrases however expands the capability of biocomputers using this general approach. Discrete inducible expression of an integrase gene and its cognate RDF can affect fully reversible changes in expression profiles in response to alternative input stimuli [105]. Essentially, this is achieved by flanking a promoter with *attB* and *attP* sites that mediate inversion of the promoter and hence expression of two alternative transcription units. Furthermore, these changes are vertically heritable and, in the absence of a reset signal, stable for over 100 generations and receptive to additional inputs for over 90 generations. In a similar fashion, the level of complexity can be scaled up by using two pairs of integrases/excisionases and inversion cassettes encoding either promoter elements or asymmetric transcription terminators. This additional “computational” capacity enables the full range of Boolean logic gates to be programmed to respond to different combinations of independent stimuli (Fig. 5) [106,107]. These capabilities raise the possibility of organisms able to not only sense their environment but also record information and respond accordingly in a predetermined manner. Potential applications could include environmental biosensing of pollutants or clinical diagnostic tests. Furthermore, it is easy to imagine up-scaling the system with additional integrases to allow more input signals to be detected or the use of orthogonal crossover sites for individual integrases (see above) to allow multiple outputs to be instigated in response to each input.

### General comments and future prospects

Phage integrases are tools for generating precise DNA rearrangements *in vivo* and *in vitro*. For many organisms or cell lines, the level of homologous recombination is extremely low and is not a viable route to generating desired genetic constructs. In these situations, the greater efficiency of site-specific recombination is an extremely useful substitute as demonstrated by the many researchers who have deployed integrases for use in higher eukaryotes. Although some integrases, notably ϕC31 and Bxb1 integrases, appear to be “plug and play” and display high levels of portability from one organism to another, others are only active in bacteria [82]. Furthermore, some serine integrases, although active and yielding the desired DNA rearrangements in eukaryotic cells, can cause small deletions in the products, *attL* or *attR* [81–83]. The eukaryotic cell environment therefore still presents a challenge for conservative site-specific recombination.

The most exciting and promising applications of phage integrases, particularly serine integrases, lie in the field of synthetic biology. These have been exemplified by their uses in metabolic pathway assembly and in the construction of digital counters and logic gates. What is needed in this field are more integrases that act orthogonally and can be multiplexed for greater versatility and increased efficiency. The use of integrases for modularising and assembling complex pathways will greatly facilitate optimization of metabolite production and allow these pathways to be easily transferred into different hosts. Moreover, the use of RDFs to retrofit metabolic pathways should also streamline the modification of previously constructed pathways. Serine integrases complement other ingenious DNA assembly methods currently available, in particular, the seamless DNA assembly methods such as Gibson assembly, SLICE and overlap extension polymerase chain reaction. It used to be the case that cloning a single gene would take at best a couple of weeks. We now hope that we can use that time more effectively in designing and reconstructing whole metabolic pathways and even whole chromosomes.

## Figures and Tables

**Table 1:** Summary of tyrosine integrases and their recombination requirements

Phage	Bacteria	*attB* (bp)	*attP* (bp)	Host factors	RDF	Length (aa)	Molecular mass (kDa)	References
λ	*Escherichia*	21	240	IHF and Fis	Xis	72	9	[108,109]
ICE*clc*	*Pseudomonas*	18	450	IHF^a^	**-**b	—	—	[26]
L5	*Mycobacterium*	29	240	mIHF	Gp36/Xis	56	6	[54,110–113]
P2	*Salmonella*	17	220	IHF	Cox	91	10	[114–117]
P22	*Salmonella*	27	260	IHF^c^	Xis	116	14	[118–121]
HP1	*Hemophilus*	18	420	IHF	Cox	79	9	[122–126]

a Not confirmed but presumed due to the presence of binding sites.

b Directionality controlled via differential integrase expression.

c Not essential for efficient recombination but binding sites present.

**Table 2:** Summary of serine integrases and their recombination requirements

Phage	Bacteria	*attB* (bp)	*attP* (bp)	RDF	Length (aa)	Molecular mass (kDa)	References
ϕC31	*Streptomyces*	34	39	gp3	244	27	[36,52]
ϕBT1	*Streptomyces*	36	48	gp3	247	28	[50,52]
TG1	*Streptomyces*	39	43	gp25	240	27	[127]
Bxb1	*Mycobacterium*	38	48	gp47	255	28	[40,51]
R4	*Streptomyces*	64	50	**-**a	—	—	[128]
ϕMR11	*Staphylococcus*	34	34	**-**a	—	—	[129]
ϕRv1	*Mycobacterium*	40	52	Rv1584c/Xis	73	8	[130]
TP901-1	*Lactococcus*	31	50	ORF7	64	8	[49,131]
A118	*Listeria*	42	50	**-**a	—	—	[132]

a Unknown at present.

## Footnotes

† http://www.norcomm.org/index.htm
